# Anatomical Computerized Exploration to Excise Malignancies in Deep Facial Compartments: An Advanced Virtual Reality Protocol for a Tailored Surgical Approach

**DOI:** 10.3389/fonc.2022.875990

**Published:** 2022-05-13

**Authors:** Alessandro Tel, Daniele Bagatto, Fabio Costa, Salvatore Sembronio, Massimo Robiony

**Affiliations:** ^1^ Department of Maxillofacial Surgery, University Hospital of Udine, Udine, Italy; ^2^ Department of Neuroradiology, University Hospital of Udine, Udine, Italy

**Keywords:** virtual surgical planning, 3D vessels, deep facial compartments, navigation, virtual endoscopy

## Abstract

**Objective/Hypothesis:**

This study describes the design and application of a novel advanced protocol for virtual three-dimensional anatomical reconstruction of the deep facial compartments, aiming to improve the preoperative understanding and the intraoperative assistance in complex resective surgeries performed for malignant diseases which extend in complex spaces, including the pterygomaxillopalatine fossa, the masticator space, and the infratemporal fossa.

**Methods:**

This study is a non-profit, retrospective, and single-institution case series. The authors clearly describe in detail imaging acquisition protocols which are suitable to segment each target, and a multilayer reconstruction technique is presented to simulate anatomical structures, with particular focus on vascular networks. Virtual surgical planning techniques are individually designed for each case to provide the most effective access to the deep facial compartments. Intraoperative guidance systems, including navigation and virtual endoscopy, are presented, and their role is analyzed.

**Results:**

The study included seven patients with malignant disease located in the deep facial compartments requiring radical resection, and all patients underwent successful application of the protocol. All lesions, except one, were subject to macroscopically radical resection. Vascular structures were identified with overall reconstruction rates superior to 90% for major caliber vessels. Prominent landmarks for virtual endoscopy were identified for each case.

**Conclusions:**

Virtual surgical planning and multilayer anatomical reconstruction are valuable methods to implement for surgeries in deep facial compartments, providing the surgeon with improved understanding of the preoperative condition and intraoperative guidance in critical phases for both open and endoscopic phases. Such techniques allow to tailor each surgical access, limiting morbidity to strictly necessary approaches to reach the disease target.

## 1 Introduction

The pterygo-maxillo-palatine fossa (PMPF), the infratemporal fossa (ITF), and the deep masticatory space (DMS) are deep facial compartments (DFC) of difficult access, due to their location, enclosed in a narrow, rigid space between the posterior wall of the maxilla and the skull base, and to the presence of densely packed vascular and nervous structures. Malignant lesions arising in such spaces can show variable infiltration of critical structures, which have to be preliminarily identified. In particular, such lesions frequently develop in critical points in which multiple vessels and nerves reciprocally cross in complex networks which are difficult to visualize using a standard two-dimensional imaging.

It is well known that in malignant tumors, the major role of surgery is to minimize the entity of a macroscopic residual disease; therefore, a wide, margin-free resection still represents the gold standard to achieve surgical radicality, allowing adjuvant therapies to maximize their effect, but at the cost of sacrificing nearby crucial structures, posing surgery at high risk of complications (1). As such, anatomical regions offer very narrow spaces for surgical maneuvers, often impairing vision; each case demands designing specific accesses. Moreover, patients presenting with diseases in DFC often present at late stages, due to a silent growing process which might cause mild disturbances, mimicking symptoms attributable to more common diseases. The result is an advanced disease, which often grows across major caliber vessels, where surgery necessarily involves a meticulous dissection of tumor tissue from arteries and veins, which in several cases have to be anticipately identified for sacrifice.

Such considerations translate in the importance to perform a preoperative, patient-specific, anatomical study as an essential step when approaching DFC, but so far, despite advances in computerized 3D reconstruction, only few incomplete reports on the virtual representation of such regions are available, which often adopt an excessively simplified approach to represent a very complex anatomy. Moreover, masses developing DFC often project within paranasal sinuses, requiring endoscopy both as a visualization improvement and as a precise tool to accomplish specific phases of surgery. Endoscopy provides a small field vision within such spaces, often in the presence of a distorted anatomy or in the presence of a major vessel concealed beneath mucosal surfaces; thus, the simulation of endoscopic approaches as part of the virtual planning is equally important, especially to ascertain the vicinity of nearby critical structures. In computerized simulation, a virtual reality animation allows to merge open surgery with endoscopic vision, replicating any endoscopic view exactly as it would appear using an optical device as the camera proceeds in depth, providing an animated sequence (2, 3).

Alongside the lack of examples for a virtual reconstruction of DFC, literature provides limited evidence on the most suitable imaging protocols that should be implemented to study diseases arising in such spaces. To address such issues, our Department has developed a novel workflow based on optimized image acquisition protocols and tailored segmentation techniques. The purpose of this study is to provide a replicable methodology to perform multilayered anatomical reconstruction of DFC, to sequentially represent structures from the skeletal backbone to finest details of vasculature and soft tissues. Moreover, the Authors show how this protocol translates into clinical applicability through intraoperative navigation and simulated endoscopy, resulting in tailored approaches optimized for each disease.

## 2 Materials and Methods

### 2.1 Patient Population/Study Design

This is an institutional review board-approved, single-institution, retrospective case series concerning the clinical application of an innovative protocol to study lesions arising in the DFC. We recruited seven patients with a malignant disease requiring radical resection located in the DFC, defined medially by the parapharyngeal space, posteriorly by a plane intersecting the clivus, anteriorly by the maxillary tuberosity, and laterally by the TMJ, including at least one between the deep lobe of the parotid gland and the lateral pterygoid muscle (**Table 1**).

**Table 1:** Characteristics of patients in relation to disease process, surgery, and protocol applicability.

ID	Sex	Age	Localization	Pathology	Surgical approach	Imaging protocol (with MR sequences)	Virtually segmented structures	Simulated procedures	Major surgical pitfalls
1	F	38	Pterygo-maxillo-palatine fossa	Adenoid cystic carcinoma	Transnasal endoscopic, transoral endoscopic, transmandibular open	CT MR 3D-VIBE T1 MR 3D-TOF MR venography	Skeletal and mucosal layer Arteries Veins Mucosal lining Tumor Masticatory muscles Parotid gland	Virtual endoscopy Mandibular swing Zygomatic flap Maxillary antrostomy	Loss of V3 and V2 (involved in radical resection) Postoperative severe limitation of mouth opening
2	M	43	Middle cranial fossa and infratemporal fossa	Anaplastic meningioma	Transcranial (neurosurgical), transzygomatic, transmandibular	CT MR 3D-VIBE T1 MR 3D-TOF MR venography	Skeletal and mucosal layer Arteries Veins Mucosal lining Tumor Masticatory muscles	Zygomatic flap Mandibular swing Craniotomy	CSF leak Meningitis Macroscopic residual neoplasm
3	M	69	Left maxilla with nasal floor erosion and extension to the DFC	Adenoid cystic carcinoma	Transoral with Jager’s jugal extended incision, endoscopic exploration	CT MR 3D-VIBE T1 MR 3D-TOF MR venography	Skeletal and mucosal layer Arteries Veins Mucosal lining Tumor Masticatory muscles	Virtual endoscopy Maxillectomy	Visible scar over the cheek
4	F	65	Deep lobe of the parotid with extension to the deep masticatory space	Mucoepidermoid carcinoma	Deep parotidectomy	CT MR 3D-VIBE T1 MR 3D-TOF MR facial nerve sequences	Skeletal and mucosal layer Arteries Parotid gland Main trunk of facial nerve	Parotid gland removal	Facial nerve resection
5	F	63	Pterygo-maxillo-palatine fossa, masticatory space	Adenocarcinoma	Transnasal endoscopic, Transmandibular open	CT MR 3D-VIBE T1 MR 3D-TOF MR venography	Skeletal and mucosal layer Arteries Mucosal lining Tumor Masticatory muscles	Virtual endoscopy Mandibular swing	Loss of V2 and V3
6	M	70	Retromandibular trigone invading the masticatory space	Squamocellular carcinoma	Transmandibular open, endoscopic exploration	CT MR 3D-VIBE T1 MR 3D-TOF MR venography	Skeletal and mucosal layer Arteries Veins Mucosal lining Tumor Masticatory muscles	Virtual endoscopy Mandibular swing	Postoperative severe limitation of mouth opening
7	M	45	Right posterior maxilla with extension to the deep masticatory space	Squamocellular carcinoma	Maxillectomy (Weber-Ferguson approach), transnasal endoscopic	CT MR 3D-VIBE T1 MR 3D-TOF	Skeletal and mucosal layer Arteries Mucosal lining Tumor Masticatory muscles	Transfacial swing, virtual endoscopy	None

### 2.2 Multilayer Imaging and Segmentation Protocol

Dedicated imaging acquisition protocols are needed to reproduce all anatomical structures for virtual planning according to a layer-by-layer model, including the skeletal framework, mucosal lining, muscles, vasculature, and lesions:

- Skeletal framework and mucosal lining: an ultrathin CT was performed with the following parameters: slice thickness = 0.625 mm, matrix = 512 × 512 px. The bony anatomy is simply segmented by using a thresholding algorithm within the bone tissue Hounsfield unit (HU) range. The resulting segmentation mask is then split into a mandible and skull-base subunit using semiautomatic techniques. The mucosal lining defined the spatial boundaries to simulate virtual endoscopy and was reconstructed from CT scan as well by applying thresholding on the density interval of the internal surface of paranasal sinuses.
- Tumor and other soft tissue: patients underwent MR with a 1.5-Tesla system (Aera; Siemens; Erlangen, Germany). After contrast medium administration, a 3D-VIBE T1-w sequence was acquired with a slice thickness of 1 mm and a matrix of 512 × 512 px.
- Arteries: to better delineate the anatomical relationships between the lesion and both extracranial portions of the internal carotid artery and external carotid artery, patients underwent 3D time-of-flight (TOF) MR with the following parameters: TR = 25.0 ms; TE = 7.15 ms; slice thickness = 0.5 mm; in-plane resolution: 0.4 × 0.4 mm; slice GAP = -25%; matrix 256 × 256 px.
- Veins: to detail venous vasculature, phase-contrast MR venography was also performed using a 2D-TOF sequence adapted for the study of posterior cranial fossa, paying particular attention to the positioning of saturation slabs and carefully avoiding any possible inflow artifacts.

**Figure 1:**
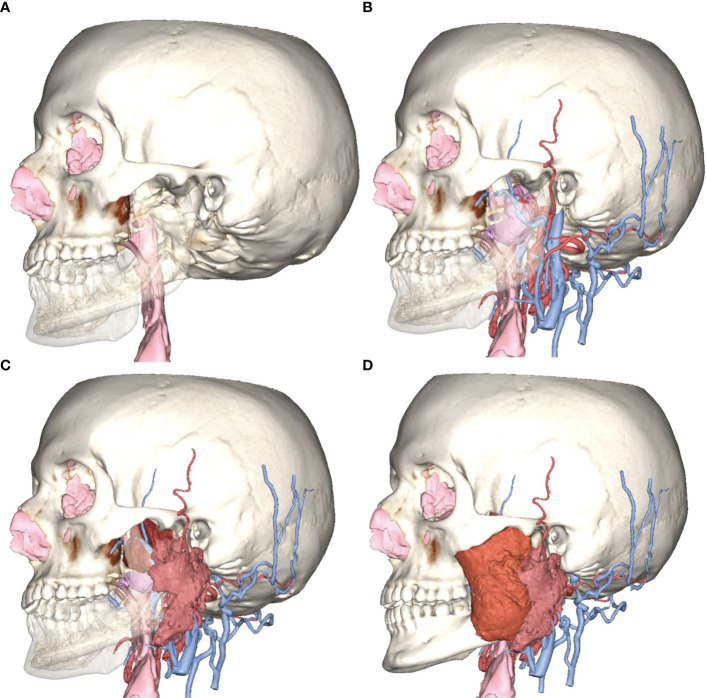
Example of multilayer anatomical reconstruction. **(A)** Skeletal and mucosal framework; **(B)** reconstruction of tumor (purple), arteries, and veins; **(C)** reconstruction of the parotid gland; and **(D)** reconstruction of masticatory muscles.

Using Materialise Mimics v24.0 (Materialise, Leuven, Belgium), CT and MR sequences were coregistered with univocal coordinates to achieve superimposition. Vessels were segmented using dynamic thresholding algorithms, which implement criteria of spatial vicinity and HU similarity to capture potentially related voxels, such as those that compose the course of a vessel. All segmentation masks were reviewed and carefully inspected for optimal correspondence with each set of DICOM images and were then re-tessellated into STL files (**Figure 1**). The result was a hierarchical tree of anatomical structures, which could be selectively inspected or hidden.

### 2.3 Virtual Surgical Planning

#### 2.3.1 Virtual Vasculature Study

**Figure 2:**
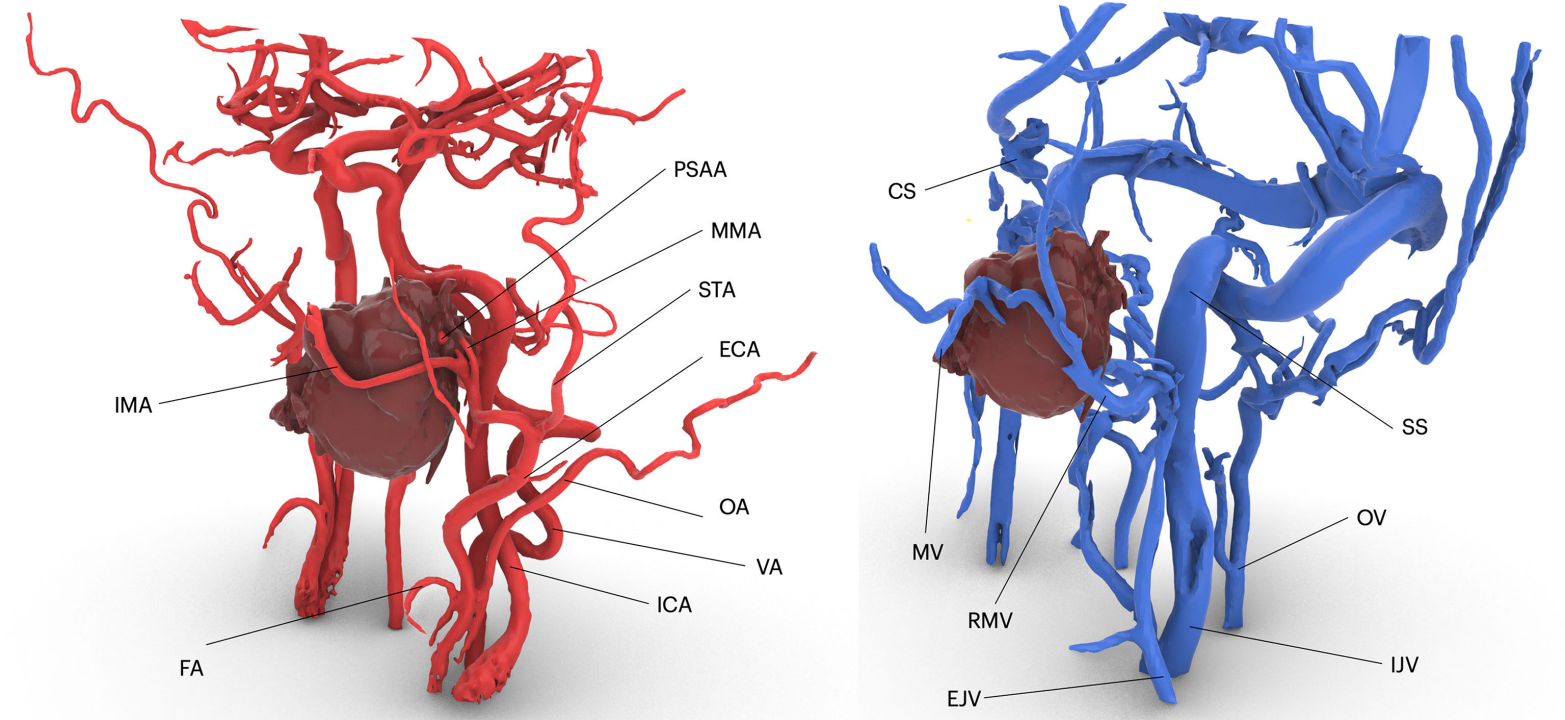
Virtual vascularization study conducted on three-dimensional models of arterial and venous vasculature, respectively derived from 3D TOF MR sequences and phase-contrast venography. IMA, internal maxillary artery; FA, facial artery; ICA, internal carotid artery; VA, vertebral artery; OA, occipital artery; ECA, external carotid artery; STA, superficial temporal artery; MMA, middle meningeal artery; PSAA, posterior superior alveolar artery; CS, cavernous sinus; M, maxillary vein; SS, sigmoid sinus; OV, occipital vein; RMV, retromandibular vein.

Vessels were virtually reconstructed according to the multilayer segmentation protocol. Vessels were mapped and named by two operators (AT and DB). In particular, operators identified the contributors to arterial flow and venous drainage located around the tumor and at least each of the major vessels: internal carotid, external carotid, internal maxillary artery, maxillary vein, retromandibular vein, and internal jugular vein (**Figure 2**). For major vessels, labels were added to the surgical planning and transferred in the navigation plan.

#### 2.3.2 Planning of Surgical Accesses in Virtual Reality

**Figure 3:**
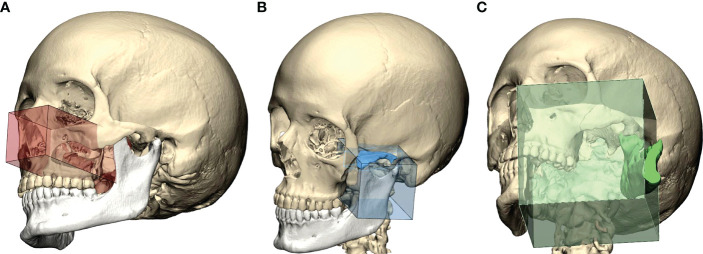
Preoperative definition of access portals for the PMPF: **(A)** anterior maxillectomy exposes the PMPF from a frontal sight, just behind the maxillary tuberosity; **(B)** transzygomatic approach raising a bone flap of zygomatic arch exposes the infratemporal fossa; **(C)** the transmandibular corridor achieved using a mandibular swing approach widens the corridor to the inferior aspect of the PMPF.

The Mimics project was imported in 3-Matic v16.0 (Materialise, Leuven, Belgium), where osteotomies were designed to simulate accesses to the deep structures. Each patient underwent individualized approach simulation depending on the location of the disease, although three common threads in virtual surgical planning were identified according to our case series:

- PMPF was exposed by simulating a mandibular swing approach, by an outward rotation of the mandibular segment across the Z-axis with a fulcrum positioned over the condyle area. The rotational movement of the mandible allowed to uncover a straightforward corridor exposing the oropharynx and the skull base.
- To access the IF, a superior-lateral transzygomatic approach yielded optimal exposure, especially if associated with a coronoidectomy and temporalis muscle disinsertion, paving the way to the lateral skull base.
- For more cranially located malignancies, especially with concomitant involvement of the paranasal sinuses, the transmaxillary portal configured a corridor for the forward exploration of the PMPF as well as the anterior skull base. The transmaxillary portal is particularly suitable for endoscopic exploration (**Figure 3**), as it develops within an empty cavity (the maxillary sinus) that offers space for surgical maneuvers without involving injuries to critical structures and reduced bleeding.

#### 2.3.3 Virtual Endoscopic Setup

**Figure 4:**
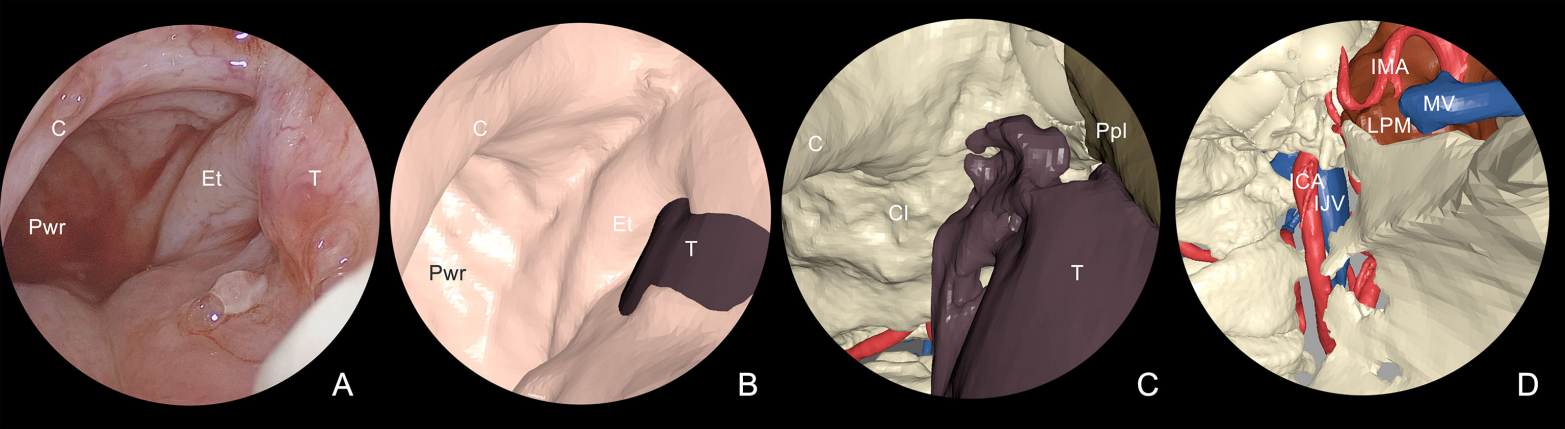
Multilayer anatomical reconstruction applied to endoscopic view. **(A)** Overview of real endoscopic scenario; **(B)** virtual endoscopy shows the mucosal lining and the tumor bulging around the tubaric orifice area; **(C)** selective hiding of the mucosa reveals the underlying tumor in relation with skeletal structures and underlying vessels; **(D)** tumor hiding reveals the proximity with dangerous structures, including IMA and ICA. T, tumor; Et, eustachian tube; C, choana; Pwr, posterior wall of the rhinopharynx; Cl, clivus; Ppl, pterygoid plate; LPM, lateral pterygoid muscle; IMA, internal maxillary artery; ICA, internal carotid artery; MV, maxillary vein; IJV, internal jugular vein.

STL files of segmented anatomy were imported into Autodesk Maya (Autodesk Inc, San Jose, CA), a comprehensive software package designed for 3D animation. A digital replica of the endoscopic optical system was simulated, and a see-through camera with defined lens parameters (focal distance: 18 mm, image refreshing rate: 50 fps) was parented to the tip of the virtual optics, allowing to scope the endonasal space with the same vision provided by the real endoscope. Surgeons virtually moved the camera within anatomical spaces and set specific key frames corresponding to prominent landmarks for each phase, which the software interpolated to yield a smooth animation sequence. Landmarks identified in nasal endoscopy were the following: head of inferior and middle turbinate, bulla, antral foramen, vidian canal, choanal opening, ethmoidal roof, pterygoid plates, Eustachian tube opening, sphenoid sinus opening, paraclival tract of ICA. Endoscopic animation was scoped for both superficial and deep layers, allowing to virtually remove mucosae to reveal underlying vasculature in critical areas around the tumor (**Figure 4**). The simulation was brought inside the operating room to be used as reference during real endoscopy.

### 2.4 Computer-Guided Surgery

#### 2.4.1 Navigation

**Figure 5:**
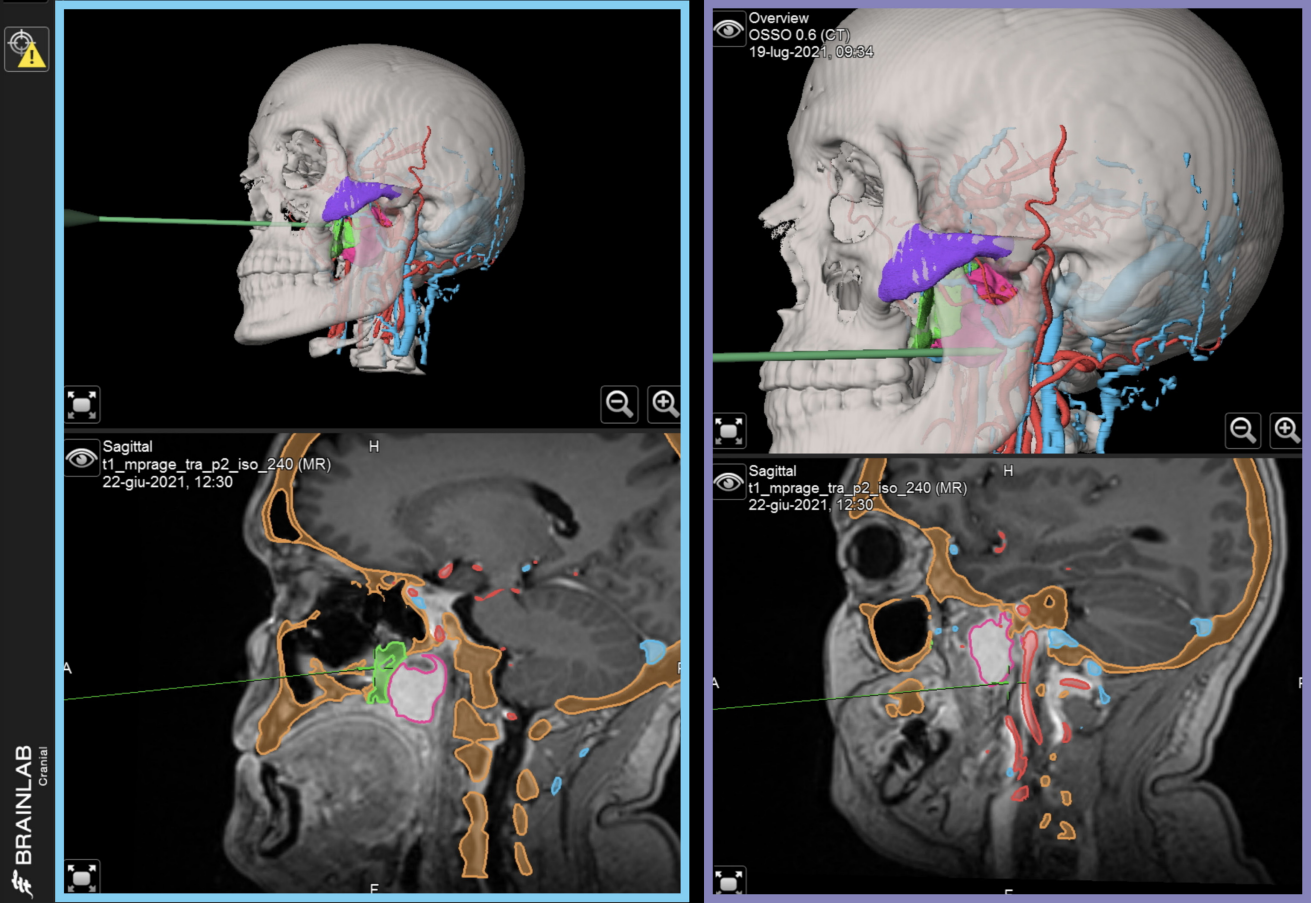
Intraoperatively navigated sequences. STL files of virtual surgical planning are navigated during surgery, providing reference for each phase. Left, blue panel: navigation of transnasal endoscopy. Right, purple panel: navigation of transoral endoscopy.

In this protocol, navigational assistance was implemented to evaluate the position of each maneuver in relation with the virtual plan. Using Brainlab Elements (Brainlab, Munich, Germany), STLs from the virtual plan were imported into the navigation project matching their position over the DICOM data, and the exported file was uploaded into the navigator. An optical stereoscopic camera was installed in the operating room, and a receiver was mounted on the patient’s head, allowing to track the probe independent of head movements required by the operators. A point-based, anatomical-landmark, recognition method was used to perform patient-to-image registration. Both phases of endoscopic and open surgery were navigated according to the virtual surgical plan (**Figure 5**).

#### 2.4.2 Surgery

Lesions arising in DFC were approached with both open and endoscopic approaches.

**Figure 6:**
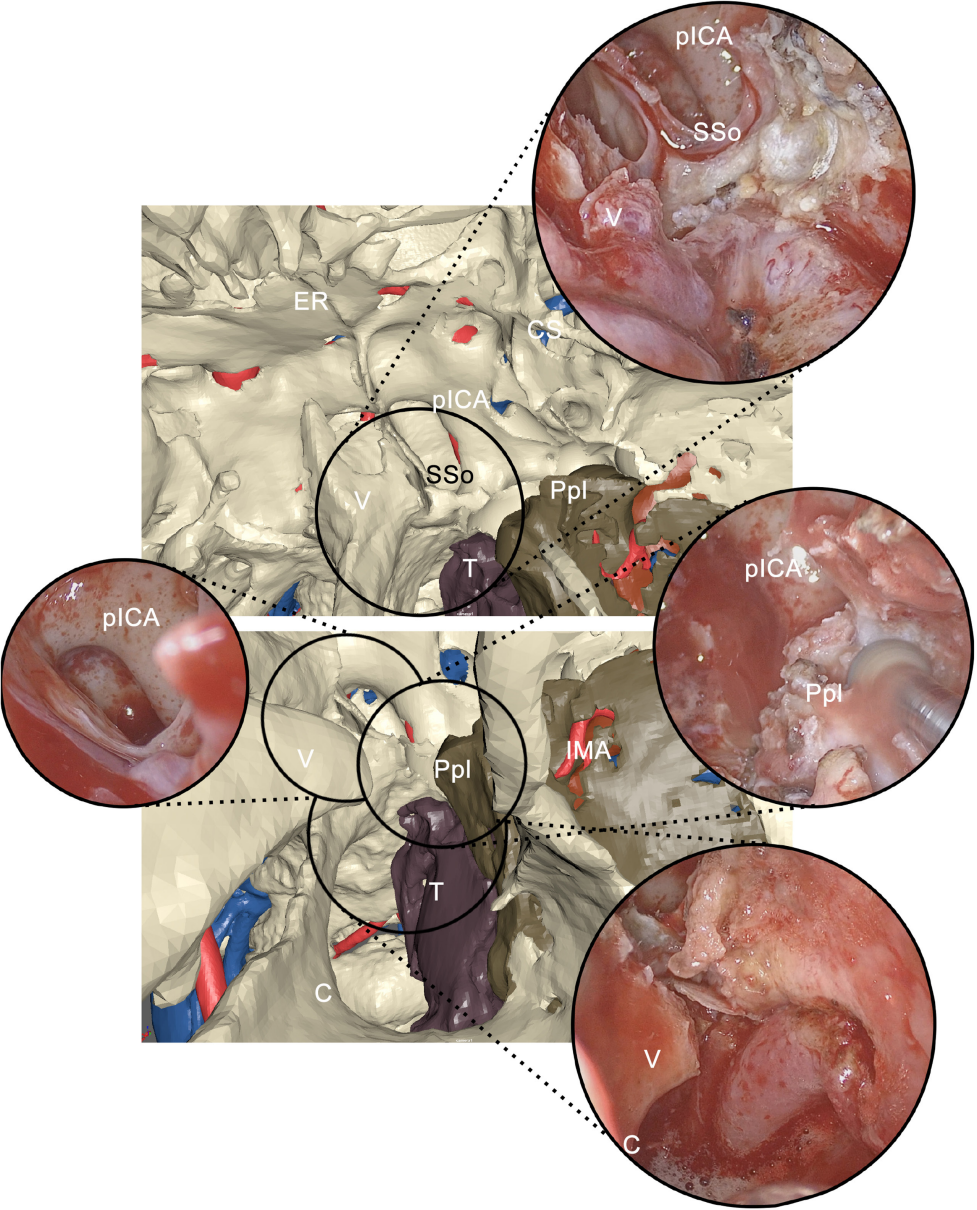
Correlation between virtual endoscopy and real endoscopy using anatomical landmarks. Virtual endoscopy allows to anticipately examine the endoscopic view. pICA, paraclival internal carotid artery; SSo, sphenoid sinus opening; CS, cavernous sinus; ER, ethmoidal roof; V, vomer; Ppl, pterygoid plate; T, tumor; C, choana; IMA, internal maxillary artery.

During transnasal endoscopy, the initial phase consisted of an inferior turbinectomy to increase space for surgical maneuvers, widening the corridor to the choanal opening. Subsequently, a medial maxillectomy was conducted extending from the antral foramen to the pterygomaxillary junction to trace the medial osteotomy line. In case of sphenoid sinus involvement, an additional phase in endoscopy was represented by widening the sinus overture using a high-speed rotating bur, scoping the intrasinusal space to reveal the underlying internal carotid artery (ICA) in its paraclival trait. Inferiorly, the emergence of the maxillary nerve and the vidian canal were systematically localized. Endoscopic osteotomies were then traced to yield mobilization of the tumor along inner bone walls; for instance, to mobilize tumors involving the PMPF, pterygoid plates were sectioned at their insertion onto the basisphenoid. As surgery progressed forward, all anatomical landmarks encountered during the virtual endoscopy were localized and related with the virtual plan (**Figure 6**).

**Figure 7:**
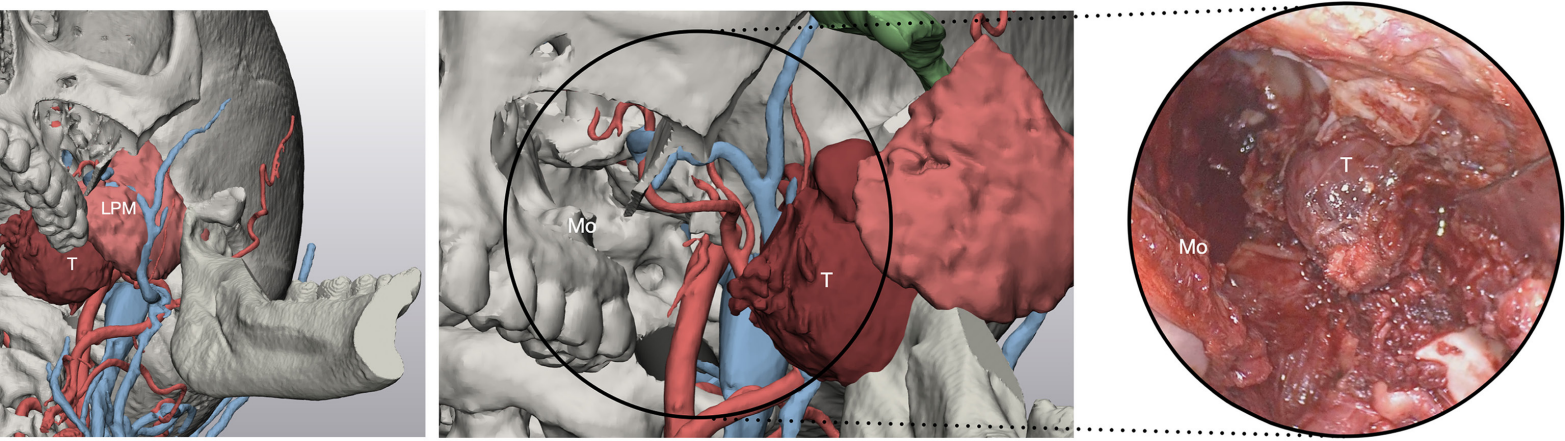
Virtual surgical planning through the transmaxillary and transmandibular portal predicts the exposure of the tumor afforded by such approaches. T, tumor; LPM, lateral pterygoid muscle; Mo, maxillary opening.

Open surgery was necessary for en-bloc resection of malignant tumors, by connecting resection margins with osteotomies traced using endoscopic approaches. PMPF was exposed by creating a transmandibular open corridor through a mandibular swing approach and a parasymphyseal osteotomy using a lip-split incision continuing with a submandibular incision. To access IF, a transzygomatic approach through zygomatic arch osteotomies was used, based on a preauricular approach and subperiosteal elevation, paying attention not to injure the frontal branch of the facial nerve crossing the zygomatic arch. Moreover, an adjunctive corridor to the PMPF was achieved through the transmaxillary route, creating a straightforward path to the retromaxillary spaces. Endoscopy was also used to check in-depth anatomy in open approaches as well, especially in the transmaxillary portal, magnifying detail and facilitating exploration of narrow spaces (**Figure 7**).

## 3 Results

All patients underwent successful application of the protocol. In three patients, it was not possible to simulate venous vessels due to the lack of MR venography.

In all patients, detailed virtual models for skeleton and mucosal lining were reconstructed from CT. Concerning the effectiveness of the protocol in detailing three-dimensional vasculature, a score of 0 was applied when it was impossible to reconstruct the vessel, 1 when reconstruction was inferior to 30% of its course, 2 when between 30% and 70%, and 3 when above 70%. In addition, an average quality score for reconstruction of each vessel was achieved by correlating individual scores with the maximum score. Major caliber vessels, including ICA, IJV, and ECA, were constantly identified and easily reconstructed in all patients, whereas inferior caliber vessels, including FA, IMA, STA, MMA, EJV, FV, RMV, and MV, were more subject to variability depending on the quality of the MR. It was possible to reconstruct at least two masticatory muscles between the masseter, internal and external pterygoid, and parotid gland in 100% of cases using 3D-VIBE T1-w sequences. The quality of reconstruction for anatomical structures is reported for each case in **Table 2**.

**Table 2:** Quality scores defining the computerized reconstruction for vascular and muscular structures.

	1	2	3	4	5	6	7	Overall quality of virtual reconstruction (%)
**Arteries**								
ICA	3	3	3	3	3	3	3	100
ECA	3	3	2	3	3	2	3	90.5
IMA	2	3	2	1	1	2	3	66.7
STA	1	2	0	0	1	0	1	23.8
MMA	0	2	1	0	2	1	2	33.3
FA	1	2	0	1	1	1	2	38.1
**Veins**								
IJV	3	3	2	3	3	2	3	90.5
EJV	2	2	1	0	2	1	0	38.1
RMV	2	3	1	0	1	2	0	42.8
MV	2	2	2	0	2	2	0	47.6
FV	1	1	0	1	0	0	1	19.1
**Masticatory muscles**	3	3	3	2	3	3	2	90.5

All surgeries were conducted using virtual endoscopy as guidance, which included the following stable, always identifiable, landmarks: the head of inferior turbinate, head of middle turbinate, choanal opening, ethmoid roof, pterygoid plates, Eustachian tube opening, and sphenoid sinus opening were successfully represented in virtual models in all cases. Other structures showed more variability in virtual reconstruction, including bulla (57%), antral foramen (85%), vidian canal (14%), and parasellar imprint of ICA (71%).

**Figure 8:**
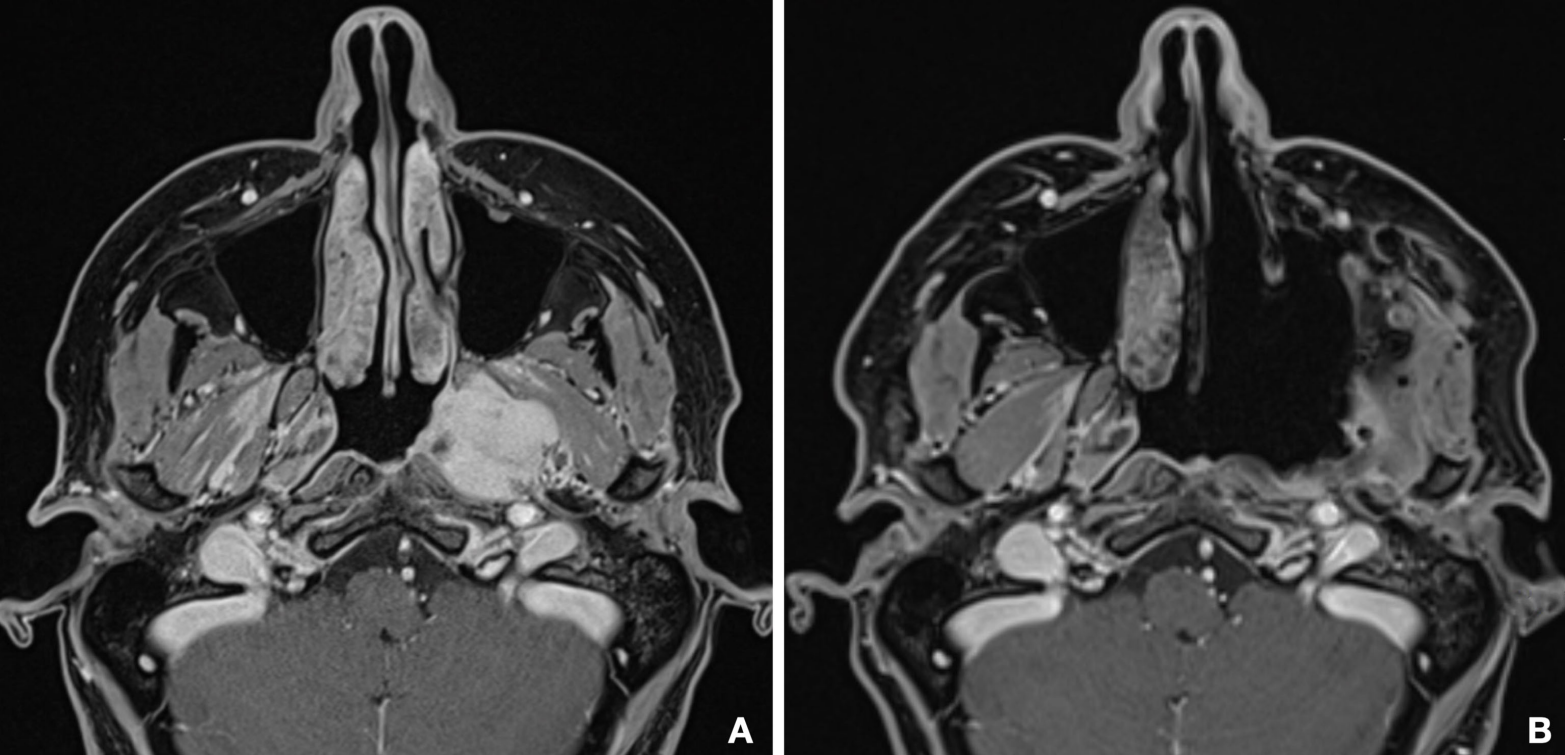
Comparison between preoperative **(A)** and postoperative **(B)** MR shows the complete emptying of the PMPF and macroscopically radical excision of the tumor.

Surgeries were successful in all cases, and lesions were subject to macroscopic radical resection, confirmed by clean margins at histology examination, except for one patient. Postoperative MR confirmed complete extirpation of PMPF and ITF in six cases (**Figure 8**), except for two patients, one case of anaplastic meningioma, whose extension in both the intracranial and extracranial space represented a limit for complete excision, and a case of adenoid cystic carcinoma, in which a residual disease was present in the posterior wall of PMPF at postoperative MR and was excised with a subsequent endoscopic procedure.

Patients undergoing surgery in the PMPF had trigeminal branches sacrificed owing to radical resection. Severe limitation of mouth opening developed in almost the totality of patients undergoing surgery in the PMPF/DMS owing to extensive muscle resection in the masticator space with subsequent scarring and radiotherapy.

The adoption of the advanced virtual reality protocol made possible to perform surgeries that in the past years were not feasible, due to the complexity of anatomical relationships with the tumor. Surgeons retrospectively reviewed the same number of cases operated for malignancies in DFC before the implementation of this protocol in a time span ranging from 2015 to 2018, as virtual-reality technologies were introduced in routine practice since 2019 at our institution. Compared with the group studied by applying the advanced computerized reconstruction, patients operated in the past showed a mean surgical time increase of 85 min (SD ± 47 min), due to phases related to tumor exposure and strategy-making, whereas in the group studied and operated using the computer-guided protocol, navigation helped to target the disease from the beginning of surgery in both endoscopic and open phases; therefore, the major benefit in terms of surgical time reduction using the computerized workflow occurred in the approach phase. Additionally, in the group operated between 2015 and 2018, four patients had macroscopic disease residual, as localization of the tumor was only performed on raw imaging data and it was not possible to interactively check the intraoperative field in relation to a virtual project using navigation. Surgeons were asked to define their approach toward surgery both before and after the introduction of the advanced virtual anatomical reconstruction, and they unanimously reported a change in mindset, with an increase in their preoperative understanding of the patient’s anatomy and diminished frequency of intraoperative imaging consultation, due to deeper understanding of the case and availability of navigation.

## 4 Discussion

Approaches to the DFC, including the PMPF, the DMS, and the ITF, represent a major challenge even for the expert surgeon, due to narrow spaces in which vulnerable vascular and nervous structures are enclosed within complex-shaped bone walls. Malignancies primarily involving these spaces or invading them as a consequence of nearby disease require difficult, multistage surgeries, in which the risk of injuring critical structures is considerable; therefore, conceptualizing in detail the patient’s anatomy before surgery is a fundamental prerequisite.

Conventional imaging techniques can define the localization of disease but fail to provide a three-dimensional representation of anatomy, especially for vascular structures, which have a tortuous course that intersects multiple spatial planes. Volume rendering, today available for many medical image software, provides accurate volumetric visualization based on DICOM data by placing specific thresholds, which succeeds in characterizing bone but does not represent soft tissues. Most of all, volume rendering-based approaches do not allow to selectively separate a specific anatomical structure and fail to provide a geometry file, which can undergo surgical planning procedures, including osteotomies and virtual endoscopy. As a consequence, unsegmented structures from raw DICOM data cannot be selectively shown or hidden, making it impossible to differentiate between venous and arterial vessels. Literature on this topic is limited, reporting only few examples of virtual anatomical reconstruction of structures through segmentation and 3D model creation. For instance, in 2021, Yang et al. interestingly described a CT-MRI image fusion-based technique to characterize tumors arising between middle cranial and infratemporal fossa: yet they managed to isolate different anatomical structures, and their reconstruction was limited to the IJV and ICA axes, with an exceedingly approximated representation of the tumor (4). A more detailed, although simplified, anatomical model to study the PPF is described by Javan and colleagues (5): the Authors designed an interactively explorable model of the PPF with attention on the limits of this space and the presence of neurovascular bundles; however, this model failed to provide a trustful representation of the actual anatomy, as it is based on simplified visualization and isolated PPF inspection, regardless of contiguous structures. Another considerable limitation of this study is that both vessels and nerves were modeled independent of their conformation on the medical images, and this step might cause inaccuracies and omissions of anatomical variations. In this study, we have shown how a very detailed vasculature study can be performed by segmenting both arterial and venous vessels directly from the patient’s imaging, enabling the virtual models to reproduce as faithfully as possible the real anatomy of a patient. Compared to the traditional two-dimensional slice-by-slice identification of vessels, three-dimensional evaluation is more immediate, allowing to inspect vessels along their spatial course, including the relationship with nearby structures and resection margins. Three-dimensional vasculature reconstruction is also important for decision making, as it can clearly represent situations in which major caliber vessels, including ECA and IJV, lie within the tumor and have to be sacrificed, allowing surgeons to anticipately know that vessel ligation can be performed, decreasing the risk of significant bleeding.

Actually, our protocol is not able to solve the issue related to cranial nerve segmentation, as nerve tracking for calibers like cranial nerves is very limited with current imaging technology. Some attempts have been made to develop imaging acquisition protocols allowing to identify tiny nervous structures in these anatomical regions: Bratbak et al. (6) described the successful identification of the pterygopalatine ganglion and the vidian canal on MR using a 3T magnet; however, not all centers own a 3T MR machine, and the sequence is not specific for nerve tracing, as for instance TOF sequences are for arteries, with the result of a very difficult and possibly inaccurate segmentation. Therefore, current imaging technology fails to provide nerve images suitable for segmentation. As a consequence, the spatial course of small caliber nerves, like the facial nerve, is still mostly deductive, according to the position of their emergence foramina, which nevertheless require a high-resolution CT scan. In particular, while the foramen rotundum and foramen ovale can be readily visualizable in almost all studies, the vidian canal, which was shown to be an important landmark to identify the anterior genu of the petrous ICA (7), should be carefully reconstructed, as shown by Fu et al. (8). Other studies focused on the utility of the infraorbital nerve as a landmark for the PMPF, hence the importance to find a reliable method to trace the nerve course (9).

As mentioned, the importance of virtual models is also related to the possibility to inspect them, simulating the same views during surgery. Concerning open surgery of the PMPF and DMS, this represents a consistent advantage, allowing to define the most adequate accesses directly on the virtual models, thus bringing the patient to surgery with a clear project in mind. By testing accesses on virtual models, the surgeon is able to define which surgical exposure is optimal for each patient, allowing to individualize surgical approaches and maximizing their effectiveness. For instance, Fahmy and colleagues conducted a study on surgical approaches by comparing exposure obtained through both open and endoscopic approaches and measured the volume of visualization yielded for each case (10). Their study was performed on two-dimensional CT data and not on virtually reconstructed segmented structures, significantly limiting the possibility to selectively manipulate objects, visualize vascularization, and perform osteotomies. Exploration of patient-specific virtual models plays a crucial role especially for endoscopic approaches, allowing to preoperatively define complex trajectories and to highlight anatomical landmarks by providing a vision totally superimposable to the real endoscopic field. The same Authors previously described a protocol to perform virtual endoscopy in orbital surgery, but this workflow is applicable to any region of the craniofacial skeleton (11). Actually, a simplified version of this protocol and suitable for the sphenoid sinus was described by Wang and colleagues (12), although the representation of critical adjacent structures, such as the ICA and the sphenopalatine artery, was roughly drafted over a volume-rendered version of the CT; conversely, our multilayer segmentation protocol allows to separately reconstruct bone, mucosa, muscles, vasculature, and tumor and to navigate between them. Virtual reality allows to identify and simulate portals which would not be visible without performing osteotomies: for instance, the incremental maxillectomy directed over the anterior maxillary wall creates an adjunctive corridor to frontally approach the PMPF (13), while a retromaxillary approach raising a zygomatic arch flap favorably exposes the infratemporal fossa (14). The possibility to simulate an outward rotation of the hemimandible with a fulcrum over the condyle allows to anticipately visualize the exposure of PMPF from an inferior sight (15). Specific transcutaneous approaches, including the Weber-Ferguson and jugal incision described by Jager (16), can be designed directly on virtual soft tissue models to aid in the choice of the most appropriate incision line to yield an exposure of the skeletal plane. Therefore, virtual planning allows to entirely customize surgical accesses, tailoring the most suitable corridor to each individual localization of disease, and it is not limited to the possibilities described in our series, but it may include in addition several transfacial swing approaches, which can be combined with endoscopy at any stage (17). Therefore, our experience is that surgeons enter the operating room having already performed a number of simulations, with a well-defined idea in mind on which accesses they should create and which sequences demolition should follow.

In this regard, especially when midface osteotomies are planned, navigational guidance allows to check the optimal tracing of bone cuts. In addition, navigation performed over virtual 3D models allows to carefully check resection margins around the tumor borders and to trace osteotomies exactly as planned (18), a consistent advantage especially for endoscopic procedures, which are not blindly performed but follow the correct path suggested by the navigator. Therefore, although navigation has been scarcely studied in the PMPF/DMS, it represents a valuable aid for surgery in the deep spaces of the face (19), especially when implemented in the setting of a reliable three-dimensional anatomical reconstruction, where it allows to interactively move within the simulated anatomy.

Virtual endoscopy can be simulated at any stage of the surgical planning, even in open surgery allowing to represent multiple scenarios which replicate the endoscopic vision encountered by the surgeon. In this respect, virtual endoscopy might also be useful to anticipately visualize the results of endoscopic exploration, establishing its correct timing within the surgical sequence and which vision correlates with a specific phase, overall improving the orientation of the surgeon.

Additionally, the whole animated sequence provides an immediate vision that, beyond the surgical purpose, is useful to convey the operative workflow to students and residents, which can start to hypothesize treatment plans in a safe environment, which takes into account the underlying anatomical complexity.

## 5 Conclusions

Despite sampling a limited number of patients, our experience suggests that the modern surgeon approaching such areas should rely upon all available instrumentation to accurately study anatomy and define a personalized treatment strategy. Virtual examination of such anatomical regions not just aids the preoperative study of patients but also provides guidance also during surgery thanks to surgical navigation and virtual endoscopy. Especially for malignancies, in which resection extends to remove the macroscopic residual of disease, the prompt identification of structures contributes to operate more safely and with an increased awareness.

## Data Availability Statement

The raw data supporting the conclusions of this article will be made available by the authors, without undue reservation.

## Ethics Statement

The studies involving human participants were reviewed and approved by the Institutional Review Board, University of Udine, # IRB_45_2020. The patients/participants provided their written informed consent to participate in this study.

## Author Contributions

AT designed the study, performed the virtual endoscopy, created the virtual models and 3D-printed models, and wrote the full paper. DB acquired the radiologic images with required protocols and wrote the imaging part of this paper. FC made the endoscopic surgery. SS read the manuscript. MR coordinated the research team and approved the final manuscript before submission. All authors contributed to the article and approved the submitted version.

## Conflict of Interest

The authors declare that the research was conducted in the absence of any commercial or financial relationships that could be construed as a potential conflict of interest.

## Publisher’s Note

All claims expressed in this article are solely those of the authors and do not necessarily represent those of their affiliated organizations, or those of the publisher, the editors and the reviewers. Any product that may be evaluated in this article, or claim that may be made by its manufacturer, is not guaranteed or endorsed by the publisher.
